# 3D-Printed Poly-Caprolactone Scaffolds Modified With Biomimetic Extracellular Matrices for Tarsal Plate Tissue Engineering

**DOI:** 10.3389/fbioe.2020.00219

**Published:** 2020-03-25

**Authors:** Liangbo Chen, Dan Yan, Nianxuan Wu, Weijie Zhang, Chenxi Yan, Qinke Yao, Christos C. Zouboulis, Hao Sun, Yao Fu

**Affiliations:** ^1^Department of Ophthalmology, Ninth People's Hospital, Shanghai Jiao Tong University School of Medicine, Shanghai, China; ^2^Shanghai Key Laboratory of Orbital Diseases and Ocular Oncology, Shanghai, China; ^3^Departments of Dermatology, Venereology, Allergology and Immunology, Dessau Medical Center, Brandenburg Medical School Theodor Fontane, Dessau, Germany

**Keywords:** 3D printing, decellularized matrix, PCL scaffold, sebocytes, tarsal plate

## Abstract

Tarsal plate regeneration has always been a challenge in the treatment of eyelid defects. The commonly used clinical treatments such as hard palate mucosa grafts cannot achieve satisfactory repair effects. Tissue engineering has been considered as a promising technology. However, tarsal plate tissue engineering is difficult to achieve due to its complex structure and lipid secretion function. Three-dimensional (3D) printing technology has played a revolutionary role in tissue engineering because it can fabricate complex scaffolds through computer aided design (CAD). In this study, it was novel in applying 3D printing technology to the fabrication of tarsal plate scaffolds using poly-caprolactone (PCL). The decellularized matrix of adipose-derived mesenchymal stromal cells (DMA) was coated on the surface of the scaffold, and its biofunction was further studied. Immortalized human SZ95 sebocytes were seeded on the scaffolds so that neutral lipids were secreted for replacing meibocytes. *In vitro* experiments revealed excellent biocompatibility of DMA-PCL scaffolds with sebocytes. *In vivo* experiments revealed excellent sebocytes proliferation on the DMA–PCL scaffolds. Meanwhile, sebocytes seeded on the scaffolds secreted abundant neutral lipid *in vitro* and *in vivo*. In conclusion, a 3D-printed PCL scaffold modified with DMA was found to be a promising substitute for tarsal plate tissue engineering.

## Introduction

The tarsal plate is one of the most important components of the eyelid. It is composed of dense connective tissue, rich elastic fibers, and a large number of meibomian glands. The tarsal plate provides both structural support and physical form, making it an essential component of the eyelid's function and appearance (Sun et al., 2015). The meibomian gland is a type of sebaceous gland with a tubuloacinar structure and holocrine function. It is located in the superior and inferior tarsal plates (Nichols et al., 2011). Meibomian glands secrete meibum, a compound made up of polar (phospholipids) and non-polar (cholesterol, wax esters, and cholesterol esters) lipids (Foulks and Bron, 2003). These lipids then diffuse onto the tear film, forming the lipid layer of the tear film, which acts to stabilize the tear film and prevent tear evaporation (Hwang et al., 2017).

The common causes of eyelid defects are mainly the tumor invasion of the tarsal plate or orbital trauma, leading to partial- or full-thickness defects of the eyelids (Zhou et al., 2010). Tarsal plate tissue engineering is vital for eyelid reconstruction, but presently it remains limited by the complexity of the tarsal plate tissue and the lack of suitable substitutes. An ideal tarsal plate substitute should have characteristics similar to the thickness, surface characteristics, strength, and flexibility of natural tarsal tissue. Moreover, it should be tissue compatible and easy to obtain and handle (Chen et al., 2005). The commonly used substitutes to repair defects of the tarsal plate mainly include hard plate mucosa (Mannor et al., 1994; Goldberg et al., 1999), nasal cartilage, and heterogenic sclera (Tenzel et al., 1975). However, these tissues have their limitations, such as large shrinkage of postoperative grafts, a limited range of tissues, and immune rejection. The biggest defect is the inability to replace the secretory function of the meibomian glands in the tarsal plate. Therefore, fabricating a tarsal plate substitute that has a certain secretion function of lipids is important.

Three-dimensional (3D) printing is a technique for creating 3D objects of an individual nature using computer-aided design (Song et al., 2018). It has played a revolutionary role in the production of tissue engineering scaffolds because it overcomes the existing limitations and creates the most suitable scaffolds through simple and effective porosity dimensions that cannot be achieved using traditional scaffolding techniques (Kao et al., 2015; Yu et al., 2016). The useful features of the additive manufacturing technology can also be further implemented in the bioprinting and bio-scaffolding of biological objects with intricate architecture (Bae et al., 2018; Hiller et al., 2018). Because of these advantages, the 3D printing technology can be very well used to print delicate and complex tissues such as printing cornea (Isaacson et al., 2018; Sorkio et al., 2018).

**Figure 1:**
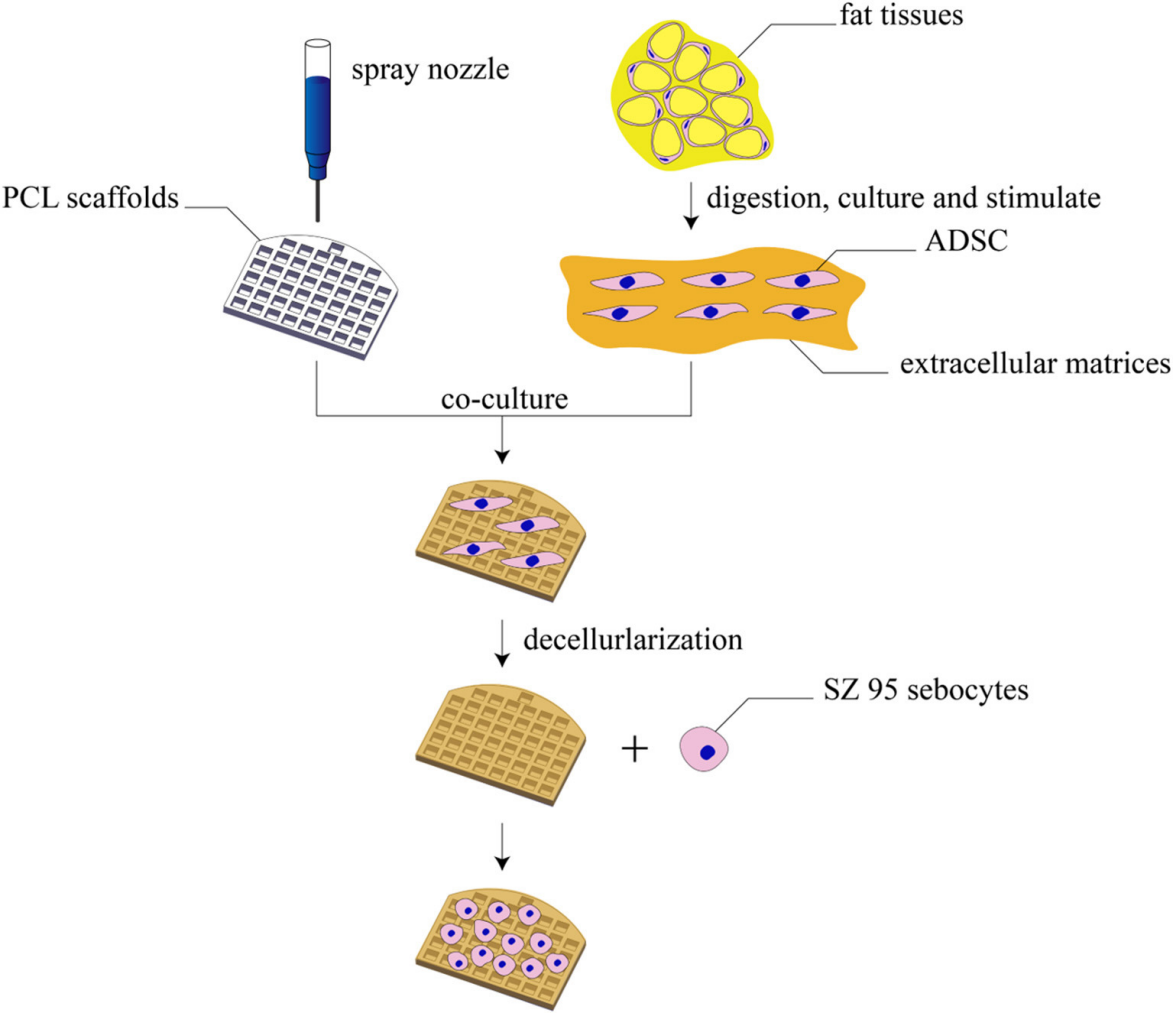
Schematic diagram of this study. The schematic diagram of fabrication of 3D-printed DMA–PCL scaffolds and seeding of SZ95 sebocytes on the scaffolds for tarsal plate tissue engineering.

In this study, the 3D printing technology was applied, for the first time, to the fabrication of tarsal plate scaffolds through PCL. Human adipose tissue–derived stem cells (hADSCs) were seeded on PCL scaffolds and then decellularized to get a DMA–PCL scaffolds so as to enhance the biological behavior. SZ95 sebocytes were seeded on the scaffold to secrete lipids. This study aimed to assess the potential value of SZ95 sebocytes seeded on DMA–PCL scaffolds in future tarsal plate tissue engineering. The extent of cytocompatibility, cell adhesion, proliferation, and adipogenesis of the scaffolds was verified *in vitro* using human SZ95 sebocytes and *in vivo* using nude mice. A schematic showing the processes used in this study is presented in Figure 1.

## Materials and Methods

### Fabrication of PCL Scaffolds

The 3D scaffolds were fabricated using a 3D printer (HTS−1200, Fochif Tech, China). The PCL beads (Mw: 40–45 kDa, supplied by Wuxi Shaxinnaxin Material, China) were put into the printing cartridge and pre-heated. Subsequently, the paste was heated to 70°C and layered with a nozzle (diameter, 0.31 μm) at a printing speed of 2 mm/s. Different pore sizes (200, 300, and 400 μm) of PCL were evaluated and an optimal pore size was chosen.

### Characterization of PCL Scaffolds

The morphology of PCL scaffolds was observed using scanning electron microscopy (SEM; JSM-6701; JEOL, Tokyo, Japan). Samples were sputtered with gold for 50 s to increase conductivity before imaging using SEM.

Mechanical properties of PCL scaffolds were determined using a uniaxial material testing machine (CMT4202, China). Rectangular-shaped specimens (20 × 10 mm) were compressed at a constant speed of 0.5 mm/min. For each sample, the maximum slope in the linear region of the compressive stress-strain curve corresponding to a strain of 0–20% was used to calculate the compressive modulus. At least three samples were tested.

### Isolation and Culture of hADSCs

hADSCs were isolated from the eyelid subcutaneous adipose tissue of young outpatients (mainly female, aged 20–25 years) admitted for blepharoplasty. This study was approved by the Institutional Review Board of Shanghai Ninth People's Hospital affiliated to the Shanghai Jiao Tong University School of Medicine. In brief, the collected adipose tissues were minced into small pieces and digested with 0.2% collagenase A for 10 h with shaking at 37°C. The cells were then centrifuged at 1200 rpm for 10 min, and the precipitated cells were cultured in Dulbecco's modified Eagle's medium (DMEM)/F12 (Invitrogen, USA) containing 10% fetal bovine serum (FBS) and 100 U/mL penicillin/streptomycin (Gibco, USA) at 37°C in the presence of 5% CO_2_. The medium was replaced every 2 days.

### DMA–PCL Scaffolds

hADSCs were cultured in DMEM/F12, containing 10% FBS and 50 μmol/L vitamin C (Sigma–Aldrich, USA) as discussed in a previous study (Ji et al., 2018). Before cell seeding, PCL scaffolds were placed in a 24-well plate. 5 × 10^4^ of hADSCs (passage 3-5) were suspended in a 50 μL culture medium, and the cell suspension was applied dropwise on top of the scaffolds. The cell-seeded scaffolds were subsequently incubated at 37°C for half an hour to allow the adhesion of the cells into the porous structure before the expansion medium was added (500 μL/scaffold). The medium was changed every 2 days for 2 weeks. After 2 weeks, the cell separation buffer was applied. The cell separation buffer was composed of 0.5% ammonium hydroxide Triton + 20 mmol/L NH_4_OH and dissolved in phosphate-buffered saline (PBS). The cell separation buffer was gradually dropped into the plate, allowed to stand for 5 min, and then washed three times with PBS. Subsequently, 100 U/mL DNase (Sigma–Aldrich, USA) was added. The scaffolds were retained at 37°C for 1 h, washed three times with PBS, dried, and stored (in the dark) at 4°C.

### Contact Angle of PCL and DMA-PCL Scaffolds

The water contact angles of PCL and DMA-PCL scaffolds were determined by the solid drop method. Measurements were handled using a goniometer, Data Physics (model OCA 15 Plus) System, equipped with an electronic syringe, a video camera and SCA 20 software. First, the scaffolds were placed on the plate of the goniometer and centered with the tip of the needle. Afterwards, using the software, a drop of 4 μL ultrapure water was released through the needle over the scaffold. The process was observed using a camera, which captured the exact moment the raindrops reached the surface of the scaffold. This procedure was repeated three times to obtain the average contact angle of the left and right parts of the droplet.

### Immunofluorescence of Collagen I and Fibronectin on PCL and DMA-PCL Scaffolds

ADSCs both with and without decellularization on scaffolds were fixed with 4% paraformaldehyde, and the samples were incubated with the following antibodies overnight at 4°C: anti-fibronectin (1:500, BD Biosciences) and anti-collagen I (1:200, Santa Cruz). Following immunofluorescence, the scaffolds were incubated with a 1:400 dilution of fluorescence-labeled secondary antibodies diluted in PBS (Alexa Fluor 488-goat anti-rabbit/mouse and Alexa Fluor 546-goat anti-rabbit/mouse, BD Biosciences) and protected from light for 1 h at room temperature. A confocal laser scanning microscope (CLSM) (A1, Nikon, Japan) was used to capture images.

### Culture of SZ95 Sebocytes and Cell Morphology on 3D Scaffolds

A total of 1 × 10^5^ SZ95 sebocytes (Zouboulis et al., 1999) were seeded on PCL scaffolds or DMA-PCL scaffolds, in DMEM (Gibco, CA, USA), supplemented with 10% FBS (Gibco), 5 ng/mL recombinant human epidermal growth factor (Peprotech, USA), and 100 U/mL penicillin/streptomycin (Gibco, USA) in a humidified atmosphere containing 5% CO_2_ at 37°C. The detailed cell culture method was the same as mentioned earlier for hADSCs. The medium was replaced every other day. Seven days after cell seeding, the scaffolds were fixed with 0.25% glutaraldehyde (Merck, Germany) at 4°C overnight. The samples were rinsed with PBS three times and then dehydrated with graded concentrations of ethanol (30, 50, 70, 80, 90, and 100% *v/v*) for 10 min each. Subsequently, the samples were critical-point dried, following which they were sputter-coated with gold and examined using an SEM.

### Live/Dead Viability Assay

Viability staining was performed using Live/Dead assay (Thermo Fisher Scientific, CA, USA) as described in a previous study (Chen et al., 2015). In brief, 5 × 10^4^ SZ95 sebocytes were cultured for 7 days in the culture medium on scaffolds or in 24-well plates (control group). After the culture solution was sucked and rinsed with PBS, the cells were incubated in PBS containing ethidium homodimer 2 (EthD-2) and calcein-acetoxymethyl ester (CAM) at 37°C for 15 min and washed with PBS for three times. Live cells were stained with green-fluorescent CAM, and dead cells were stained with red-fluorescent EthD-2. A fluorescent microscope (Olympus BX51; Olympus, Tokyo, Japan) was used to capture images of cell staining.

### Quantification of Cell Viability

To detect the effect of 3D-printed scaffolds with or without DMA on cell proliferation, a cell CCK-8 assay kit (Dojindo, Japan) was used. In brief, SZ95 sebocytes were seeded onto the scaffolds at a density of 2 × 10^4^ cells/well in 24-well plates. After 0, 1, 3, 5, and 7 days of cell seeding, the cells were incubated with 10% CCK-8 in the medium. After incubation for 4 h, the absorbance of each well was measured at 450 nm using a microplate reader (ELx800, Bio-Tek, USA).

### Immunofluorescence of F-actin and Assessment of Lipid Production on Scaffolds

SZ95 sebocytes were seeded onto the scaffolds at a density of 5 × 10^4^ cells/well in 24-well plates. After 7 days of culture, the SZ95 sebocytes were fixed with 4% paraformaldehyde (Sigma–Aldrich), dissolved in PBS for 15 min, and washed with PBS at room temperature. The specimens were immersed in phalloidin conjugated to Alexa Fluor 505 (1:1,000 dilution in PBS, Invitrogen) to obtain an F-actin cytoskeleton for 1 h at room temperature. Then, the scaffolds were washed with PBS for three times. For neutral lipid droplet staining, the cells were incubated in HCS LipidTox solution (1:1,000 dilution in PBS, Invitrogen) for 30 min at room temperature. CLSM was used to capture images of cell staining.

### RNA Isolation, Reverse Transcription, and Quantitative Polymerase Chain Reaction

The SZ95 sebocytes were seeded onto the scaffolds at a density of 5 × 10^4^ cells/well in 24-well plates. After 7 days of culture, the cell-loaded scaffolds were taken out from the culture dish using microscope forceps and immersed in TRIzol reagent (Thermo Fisher Scientific, USA). Total RNA was extracted using TRIzol reagent following the manufacturer's protocols. The RNA concentration was measured using a spectrophotometer and NanoDrop 2000 software, and the OD260/280 ratio of RNA was considered to be of purity between 1.9 and 2.1. Subsequently, 1,000 ng RNA was reverse-transcribed using a PrimeScript RT reagent kit (TaKaRa, Japan). A real-time polymerase chain reaction (PCR) was performed using a 7500 Real-Time PCR Detection System (Applied Biosystems). After 40 cycles of amplification, the relative mRNA was analyzed using the Pfaffl method (Pfaffl, 2001). The PCR efficiency of the reaction was measured with primers using serial dilutions of cDNA (1:1, 1:5, 1:25, 1:125, 1:625, and 1:3125). The primer sequences used for real-time PCR are listed in Table 1.

**Table 1:** Primers used for quantitative polymerase chain reaction.

**Gene**	**Forward (5^′^-3^′^)**	**Reverse (5-3^′^)**	**Annealing temperature (**°**C)**	**Product size (bp)**
PPARγ	ACCCAGAAAGCGATTCCTTCA	GTGTCAACCATGGTCATTTCTTGT	60	70
Ki67	TGAGCCTGTACGGCTAAAACA	CACTCTCATCAGGGTCAGAAGA	60	179
ZO-1	TTCTGAGGCCTGTAACCATTT	AATTGGATACCACTGGGCATAC	60	245
IL-6	TTCGGTACATCCTCGACG	CAGGCAAGTCTCCTCATT	60	167
Caspase-3	CATGGAAGCGAATCAATGGACT	CTGTACCAGACCGAGATGTCA	60	139
SREBP-1	GGAGCCATGGATTGCACTTT	TCAAATAGGCCAGGGAAGTCA	60	77
FAS	GACCGCTTCCGAGATTCC	GATGGCAGTCAGGCTCAC	60	137
FADS-2	TGTCTACAGAAAACCCAAGTGG	TGTGGAAGATGTTAGGCTTGG	60	128
GAPDH	TCGGAGTCAACGGATTTGGT	TTCCCGTTCTCAGCCTTGAC	60	181

### *In vivo* Implantation

Six nude mice were supplied by the Shanghai Animal Experimental Center. All procedures on animals were performed according to the Guidelines for Care and Use of Laboratory Animals of Shanghai Jiao Tong University School of Medicine and approved by the Animal Ethics Committee of Shanghai Ninth People's Hospital affiliated to Shanghai Jiao Tong University School of Medicine. 1 × 10^6^ sebocytes were seeded on 3D scaffolds and cultured for 7 days *in vitro*. Then, the cell-seeded scaffolds were implanted into the nude mice subcutaneously. After implantation for 1 month, the nude mice were sacrificed and the samples were collected for further experiments. Each experiment was tested in triplicate. The implants were embedded in optimal cutting temperature compound (Sakura Seiki, Tokyo, Japan) and then cut into 8-mm-thick sections. Human SZ95 sebocytes were stained with human nuclear antigen antibody (Novus Biological, USA, 235-1) to evaluate the *in vivo* proliferation status. For Oil Red O staining, frozen DMA-PCL scaffold sections were fixed in 4% paraformaldehyde for 15 min, washed in PBS for 5 min, and stained for 10 min in freshly prepared Oil Red O solution. After washing with PBS for 15 min, the sections were counterstained with hematoxylin and mounted in 90% glycerol.

### Statistical Analysis

A computer-based Sigma Gel System (SPSS Inc., IL, USA) and the Image J program were used to analyze the integral optical density (IOD) of immunofluorescence images. A one-way analysis of variance followed by the Student *t*-test was used to determine the statistical significance (*P-*value) of the obtained data. All data presented in this study were reported as the mean ± SEM of three parallel studies. A *P* < 0.05 was considered statistically significant (^*^P < 0.05 and ^***^P < 0.001).

## Results

### Characterizations of 3D-printed Scaffolds

**Figure 2:**
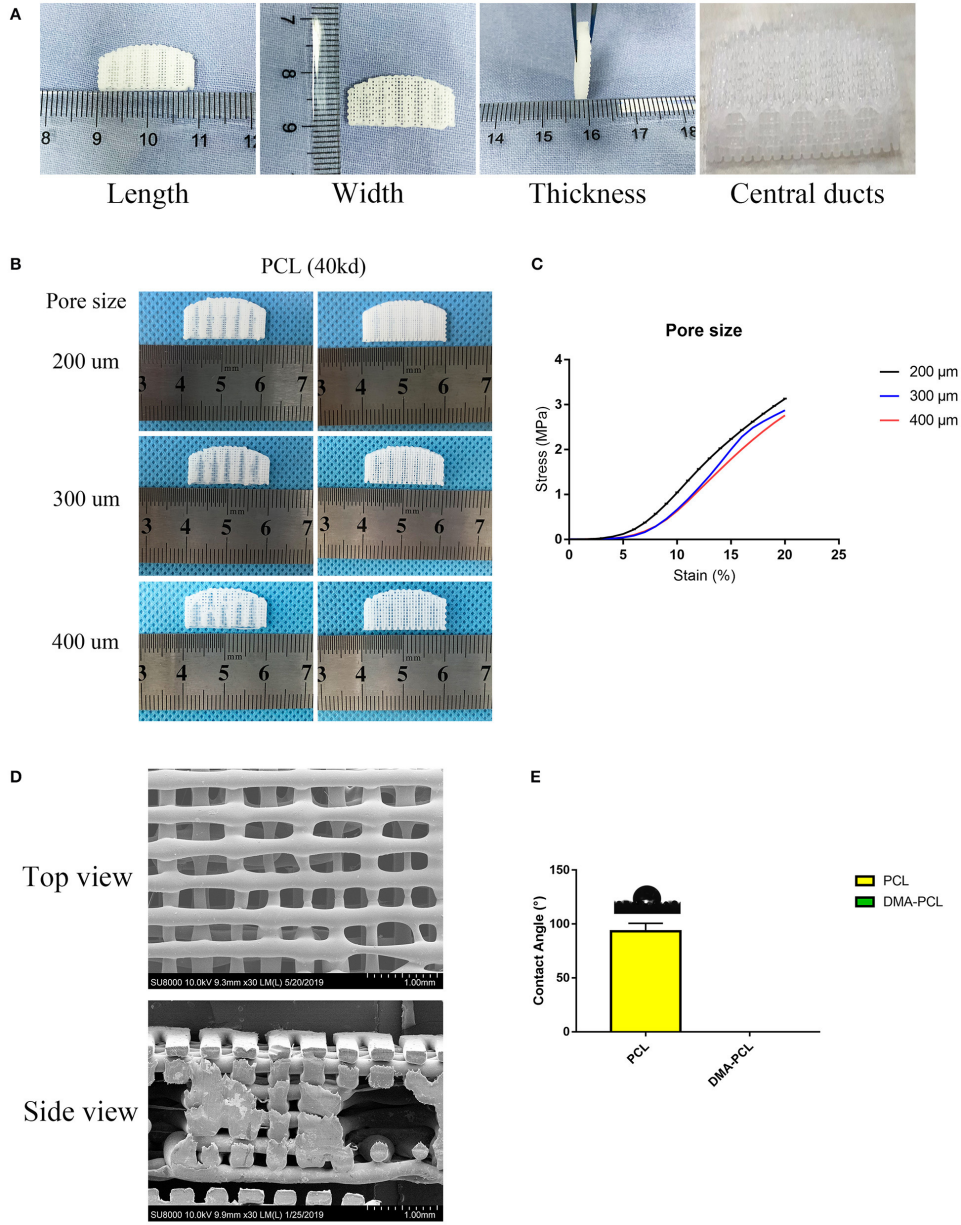
3D-printed PCL scaffold and its detailed parameters. **(A)** Length, width and thickness (20 × 10 × 2 mm) of 3D-printed scaffolds were presented, with five central ducts (diameter, 1 mm) inside the scaffold. **(B)** Different printing pore sizes (200, 300, and 400 μm) were printed and shown. Central ducts were well-formed when the printing pore size was set to 200 or 300 μm. In contrast, when the pore size increased to 400 μm, the central ducts were not well formed due to the collapse of some areas. **(C)** Typical compressive stress-strain curves of different pore sizes of 3D-printed PCL scaffolds were demonstrated. Compressive modulus decreased when the pore size increased from 200 to 400 μm. **(D)** SEM images showed the top view and side view of 3D-printed scaffolds with a pore size of 200 μm. **(E)** Contact angle of water on PCL and DMA-PCL scaffolds. Photograph of the drop at the exact moment it reached the sample surface. The mean contact angle of water on PCL scaffolds was 93.3 ± 7.5°, while the water drop was immediately absorbed into the DMA-PCL scaffolds.

The parameters of this scaffold were based on the study by Dr. Michelle (Sun et al., 2015), who examined 10 healthy tarsal plate tissues. The 3D scaffolds measured 20 × 10 × 2 mm, with five central ducts (diameter, 1 mm) inside the scaffold (Figure 2A).

Printing pore size of 200, 300, and 400 μm were used (Figure 2B). When the pore size of the printing scaffold was set to 400 μm, the central ducts were not well-formed because the spacing of the central duct was too large. When the printing pore size was set to 200 or 300 μm, the shape of the central duct was well-formed. However, considering that the smaller the pore size, the larger the area of the cell for cell attachment, the more the cells proliferated in the duct. The compressive strength was measured to confirm the operability of the scaffolds in tissue engineering. Typical compressive stress-strain curves of different pore sizes of 3D-printed PCL scaffolds were shown (Figure 2C). The compressive modulus decreased slightly when the pore size increased from 200 to 400 μm. According to a previous study, the mean initial Young's modulus of the human tarsal plate was 0.14 MPa (Sun et al., 2015). The printing scaffolds (200 μm) presented a mean compressive modulus of 0.23 MPa, which was not particularly different from that of the human tarsal plate. Therefore, considering the structure and the strength of the scaffolds, 200 μm was chosen as the optimal printing parameter in this study.

Figure 2D shows the SEM image of the scaffolds with a pore size of 200 μm. The diameter of the wire and the pore size were found to be the same, and the central duct was also well formed.

Material's wettability is an important parameter affecting the attachment, proliferation, migration, and viability of cells (Yao et al., 2017). The water contact angle was measured to determine the wettability of the scaffolds. The results revealed that the average water contact angle of the PCL scaffold was 93.3 ± 7.5°, resulting in hydrophobic surfaces (Figure 2E). The water drop in the DMA-PCL scaffolds was immediately absorbed into the scaffolds, indicating they were hydrophilic. However, because water droplets penetrated the surface too quickly for the camera to take a picture, the result was presented as a Supplementary Video 1.

### Evaluation of DMA and Morphology of SZ95 Sebocytes on DMA–PCL Scaffolds

**Figure 3:**
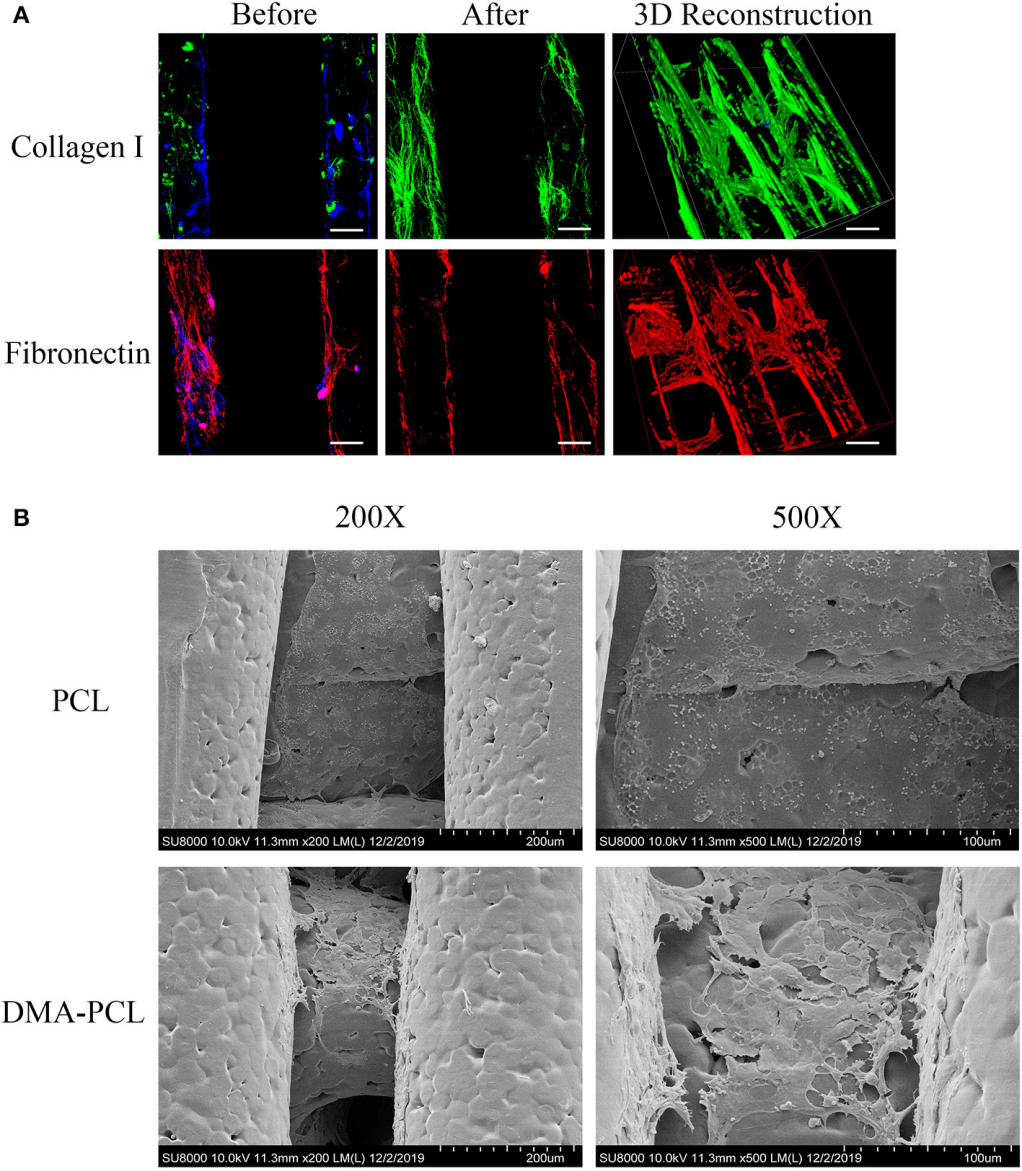
Evaluation of DMA and morphology of SZ95 sebocytes on PCL and DMA–PCL scaffolds. **(A)** Representative immunofluorescence staining images of collagen I and fibronectin. Both matrix proteins were immunostained with strong fluorescence intensity on DMA-PCL scaffolds (scale bar: 200 μm). Immunofluorescence was used to detect the expression of collagen I (green) and fibronectin (red) after hADSCs were seeded on the scaffold, before and after the decellularized matrix. After the decellularized matrix, the nucleus (4′,6-diamidino-2-phenylindole, DAPI, Blue) disappeared, and collagen I and fibronectin formed a uniform fiber network structure on the surface of the scaffold. **(B)** SEM images showed the morphology of SZ95 sebocytes 7 days after seeding on PCL and DMA-PCL scaffolds. Compared with the PCL scaffold, the cells on the DMA-PCL scaffold showed tighter cell connections, and the cell morphology was more complete.

The partial composition of DMA was revealed using immunofluorescence staining of collagen I and fibronectin. As shown in Figure 3A, both matrix proteins of collagen I and fibronectin were immunostained with strong fluorescence intensity, indicating that the surface of the scaffold was covered with sufficient DMA. The ideal material scaffolds for tissue engineering ought to maintain a normal morphology and proliferation of the cells. The cells with a cobblestone-like morphology adhered to, and were spread on, the scaffolds 7 days after SZ95 sebocytes were seeded on DMA–PCL scaffolds (Figure 3B). Compared with the PCL scaffold, the cells on the DMA-PCL scaffold showed tighter cell connections and the cell morphology was more complete.

### Cell Viability and Proliferation

**Figure 4:**
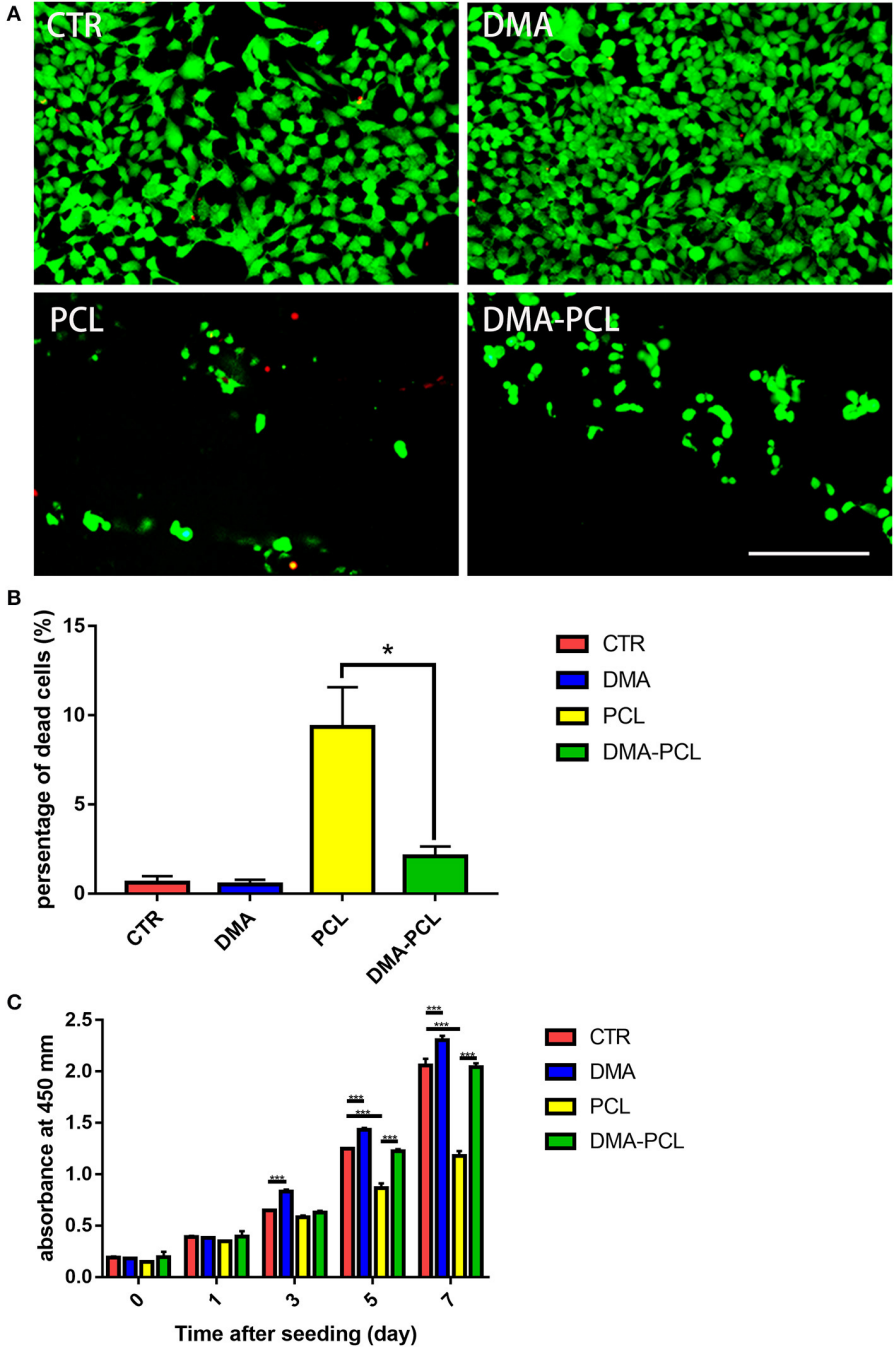
Cell viability and proliferation on DMA-PCL scaffolds. **(A)** Viable cells on the DMA–PCL scaffolds were evaluated using a Live/Dead staining assay. The live cells were stained green, and the dead cells were stained red (scale bar: 100 μm). **(B)** Statistical results of the Live/Dead staining assay showed that the proportion of dead cells on PCL scaffolds was significantly higher than that on the culture dish, while the proportion of dead cells of DMA-PCL dropped significantly. **(C)** Proliferation of SZ95 sebocytes on DMA-PCL scaffolds 0, 1, 3, 5, and 7 days after cell seeding were determined using a CCK-8 assay. DMA coated on the PCL scaffolds could significantly enhance the proliferation of sebocytes compared with the PCL group. Values are expressed as mean ± standard deviation. *P < 0.05 and ***P < 0.001. CTR, Culture dish; DMA, culture dish coated with DMA; PCL, poly-caprolactone scaffold; DMA-PCL, poly-caprolactone scaffold coated with DMA.

A Live/Dead assay kit was used to evaluate the biocompatibility of the scaffold. The biocompatibility of the scaffold was significantly increased by coating it with DMA, as shown in Figure 4A. Figure 4B shows statistics pertaining to Live/Dead results: the proportion of dead cells on PCL scaffolds was significantly higher than that on culture dish or DMA-coated culture dish, while the proportion of dead cells of DMA-PCL dropped significantly. The results of CCK-8 assay were shown in Figure 4C. It was observed that from day 3, the OD 450 value in the DMA group was significantly higher than that in the CTR group, which proved that DMA could promote the proliferation of sebocytes. Meanwhile, the OD 450 value in the PCL group was significantly lower than that in the other three groups on days 5 and 7, demonstrating that the simple PCL scaffolds were not conducive to cell proliferation. Compared with the PCL group, the OD 450 value in the DMA-PCL group significantly increased, confirming that DMA layer on the PCL scaffold promoted sebocyte proliferation as well.

### Lipid Synthesis on PCL and DMA–PCL Scaffolds

**Figure 5:**
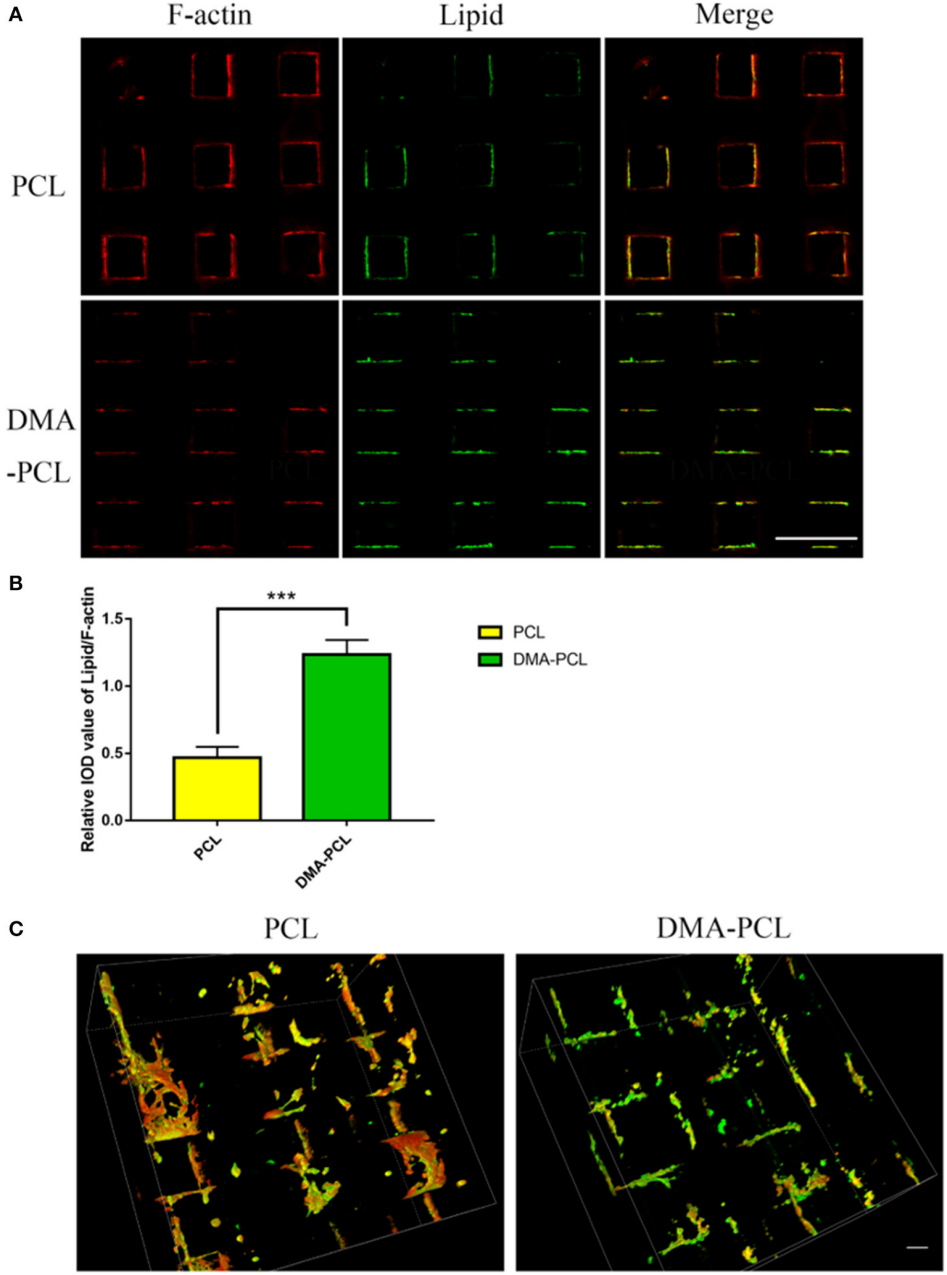
Comparison of lipid droplet formation on PCL and DMA-PCL scaffolds. **(A)** Representative immunofluorescent staining images of F-actin and neutral lipid after cells were seeded 7 days *in vitro* on PCL and DMA-PCL scaffolds (scale bar: 500 μm). Significantly higher amounts of lipids were secreted by sebocytes on the DMA-PCL scaffolds compared with that on the PCL scaffolds. **(B)** Quantitative analysis of the relative IOD value of Lipid/F-actin. **(C)** 3D volume image of F-actin and lipid 7 days after cell seeding. The amount of lipids secreted by sebocytes on DMA-PCL scaffolds was obviously higher than that on PCL scaffolds (scale bar: 100 μm). ***P < 0.001.

SZ95 sebocytes were cultured on PCL and DMA–PCL scaffolds for 7 days and then stained with F-actin and LipidTox. F-actin reflected the number of cells on scaffolds. Therefore, the relative IOD of Lipid/F-actin might partially reflect the cells' capacity to secrete lipids. Figure 5A showed the immunofluorescence of F-actin and lipid on PCL and DMA-PCL scaffold, and Figure 5B showed the statistical results of lipid synthesis. Figure 5C showed a 3D reconstruction of lipid on PCL scaffolds. SZ95 sebocytes seeded on DMA–PCL scaffolds secreted more lipids than those seeded on PCL scaffolds (*P* < 0.001), indicating that DMA could promote the differentiation of SZ95 sebocytes and contribute to the adipogenesis function.

### Gene Expression

**Figure 6:**
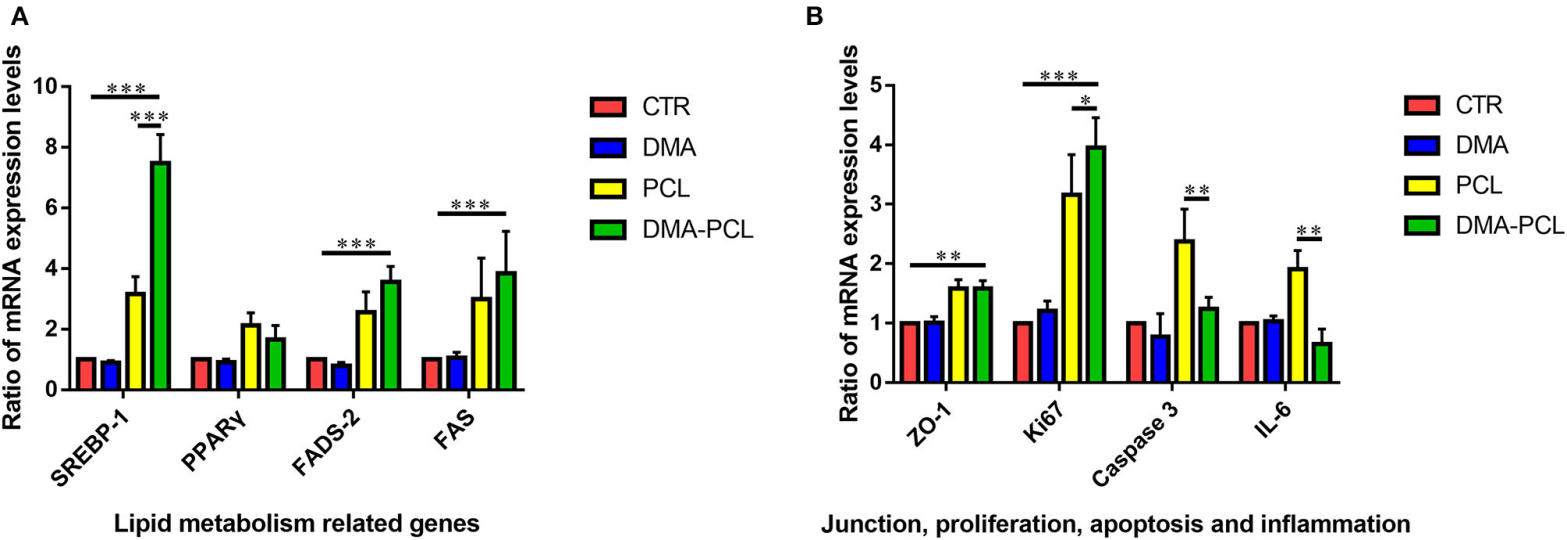
Gene expression of cells after seeding on PCL and DMA–PCL scaffolds for 7 days. **(A)** Expression levels of four lipid metabolism genes (SREBP-1, PPARγ, FADS-2, and FAS) were tested. Expressions levels of SREBP-1, FADS-2 and FAS were significantly higher in cells on DMA-PCL scaffolds indicating 3D-printed scaffolds better promoted lipid synthesis. **(B)** Expression levels of ZO-1 (a junction gene), Ki67 (a proliferation gene), Caspase-3 (an apoptosis-related gene), and IL-6 (an inflammation cytokine). The expression levels of ZO-1 and Ki67 were significantly higher in cells on DMA-PCL scaffolds compared with those in cells on CTR, indicating that DMA-PCL enhanced the adhesion and proliferation of sebocytes. Meanwhile, the expression levels of Caspase-3 and IL-6 were significantly lower on the DMA-PCL scaffold compared with the PCL scaffold, indicating that DMA coated on the PCL suppressed apoptosis and inflammation. SREBP-1, Sterol-Regulatory Element Binding Protein-1; PPARγ, Peroxisome Proliferator-Activated Receptor-γ; FADS-2, Fatty Acid Desaturase-2; FAS, Fatty Acid Synthase; ZO-1, Zona Occludens-1; IL-6, Interleukin-6. *P < 0.05, **P < 0.01, and ***P < 0.001.

The *in vitro* lipid metabolism related gene expression assay (Figure 6A) clearly indicated that SZ95 sebocytes cultured on DMA-PCL scaffolds exhibited significantly higher expression of SREBP-1, FADS-2 and FAS genes after 7 days compared with cells cultured on PCL scaffolds and on culture wells. SREBP-1, FADS-2, PPARγ, and FAS expressed on 3D scaffolds were at least two-fold of those expressed on culture wells. Also, DMA-PCL had higher gene expression levels for SREBP-1, FADS-2, and FAS compared with PCL, indicating that, perhaps, DMA was crucial during the process of sebocyte lipid metabolism. Meanwhile, the expression levels of genes related to cell proliferation, adhesion, apoptosis, and inflammation were examined (Figure 6B). The expression level of Ki67 on 3D scaffolds were at least three-fold of that on culture wells. Although the expression levels of IL-6 and caspase-3 in cells on PCL scaffolds were higher compared with cells that were cultured on culture wells, the expression levels of these two genes significantly decreased after DMA was coated on the scaffolds. These results further demonstrated that DMA played an important role in promoting cell proliferation and exhibiting an anti-inflammatory effect at the RNA level.

### *In vivo* Status of SZ95 Sebocytes

**Figure 7:**
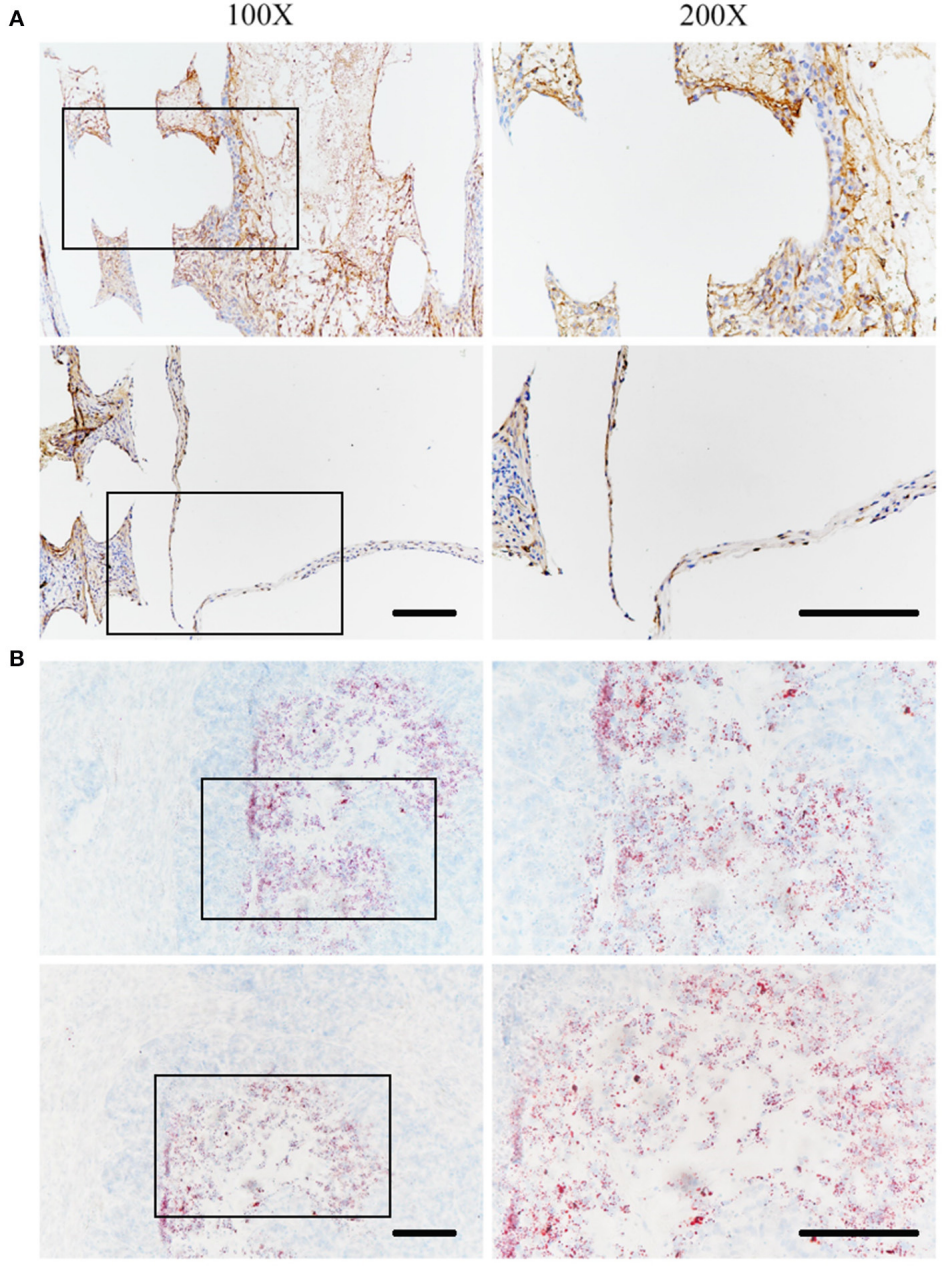
*In vivo* status of SZ95 sebocytes. SZ95 sebocytes were seeded on DMA-PCL scaffolds, and the cell-scaffold complex was implanted into the nude mice subcutaneously. **(A)** Immunohistochemistry staining images for human nuclear antigen antibody. Sebocytes on the scaffolds still proliferated well *in vivo* after 1 month. A meibomian gland acinar like structure following the shape of the scaffold could be observed, together with an intact epithelial layer. **(B)** Oil Red O staining image. Amount of lipids were secreted by sebocytes inside the scaffolds *in vivo* (scale bar: 200 μm).

The *in vivo* status of SZ95 sebocytes was observed using immunohistochemistry 1 month after the scaffolds were embedded under the skin of nude mice. The sebocytes proliferated well *in vivo* after 1 month and formed a meibomian gland acinar-like structure with an intact epithelial layer (Figure 7A). At the same time, the scaffold with sebocytes was stained with Oil Red O. The scaffold was found to have wild lipid distribution, which proved that the sebocytes still secreted abundant lipids *in vivo* (Figure 7B).

## Discussion

Tarsal plate tissue engineering has been difficult to achieve due to the complex structure and lipid secretion function of tarsal plate. In this study, 3D printing technology was used, for the first time, to fabricate tarsal plate scaffolds. Meanwhile, the surface of the scaffold was coated with DMA, which could be simply achieved by seeding autologous ADSCs. The as-prepared DMA–PCL scaffolds showed good biocompatibility and enhanced cell proliferation, differentiation, and anti-inflammation characteristics. Sebocytes seeded on DMA–PCL scaffolds secreted abundant lipids *in vitro* and proliferated well *in vivo*.

Few studies have been conducted to date in the field of tarsal plate engineering. In 2010, Dr. Jing Zhou used poly(3-hydroxybutyrate-*co*-3-hydroxyhexanoate) to fabricate scaffolds and applied them to the rat eyelid tarsal plate defects. The morphology of the eyelids was repaired 2 months after implantation (Zhou et al., 2010). In 2018 Dai et al. established a poly(lactide-*co*-glycolide)/fibrin gel/bone marrow stem cells/ (lipofectamine/pDNA-transforming growth factor-β1) construct and applied it to a rabbit eyelid tarsal plate defect model. After 8 weeks, a new eyelid tarsal plate was generated (Dai et al., 2019). The shape of the eyelids was successfully regenerated in these studies, but the function of secretion of lipid by meibomian glands could not be restored.

Therefore, this study was conducted to mimic the structure of the tarsal plate, simultaneously focusing on the function of lipid secretion.

Among the synthetic polymers, PCL has attracted extensive attention because of its biocompatibility, low melting point (59–64°C), good solubility, and ease of use (Bahcecioglu et al., 2019). It was widely applied in 3D printing systems involving selective laser sintering and FDM. Its widespread adoption could be partially attributed to its regulatory approval as a synthetic polymer for tissue repair and as a drug delivery vehicle by the US Food and Drug Administration (Jing et al., 2018). As a result, the scaffold printed with PCL was easier to apply in the clinic. In this study, PCL was used to fabricate a tarsal plate scaffold using the FDM technique. Compared with the traditional processes, such as particle/salt leaching (Esposito et al., 2013) and electrospinning (Baker and, 2007), FDM was a rapid prototyping technique that could produce a 3D scaffold with a complete control of geometric parameters, such as pore size, porosity, and pore interconnection size (Lantada and Morgado, 2012). As mentioned earlier, the shape of the eyelid defect caused by the tumor resection or trauma was irregular in many cases. 3D printing technology could accurately print the bionic tarsal plate according to the shape of the eyelid defect. Meanwhile, according to clinical experience, tarsal plate regeneration is hard and more time-consuming. PCL degrades considerably more slowly compared with other aliphatic polyesters *in vivo* (Kweon et al., 2003), and the scaffold can maintain the initial mechanical support until adequate tissue ingrowth occurs (Zhang et al., 2017). Therefore, the relatively slow degradation of PCL could provide mechanical support for enough time.

Of course, PCL also has some disadvantages, such as poor hydrophilicity and poor cell adhesion (Sun et al., 2006). Hence, most of the PCL-based tissue constructs fail to mimic the biochemical characteristics of native tissues. Extracellular matrix (ECM) can provide cells with a complex cellular environment and maintain an ideal structure for cells at both the tissue and organ levels (Wu et al., 2019). As reported, decellularized ECM has discrete influences on matrix assembly, cell proliferation, migration, signaling, and cellular feedbacks compared with planar protein-coated surfaces (Satyam et al., 2020). Therefore, decellularized matrix technology has been used to improve the biocompatibility of PCL. For example Guilak et al., showed that decellularized ECM from biological tissues could significantly enhance wound healing, even in chronic-inflammation-prone diabetic rat models (Guilak et al., 2009). Ji et al. discovered that the DMA enhanced retinal progenitor cell proliferation and neuronal differentiation (Ji et al., 2018). In this study, autologous ADSCs were used for decellularization. On the one hand, the adipose tissue was abundant and superficial, which could be harvested without causing much damage to the body compared with other tissues. On the other hand, some relative researches on the function of DMA were already done in the lab. Thus, the DMA-PCL scaffolds could be easily achieved and the effect of DMA on the adhesion and proliferation of SZ95 sebocytes was investigated. The data visibly demonstrated that DMA enhanced cell adhesion and proliferation, probably due to the enhanced hydrophilicity of PCL scaffolds (Pattison et al., 2005; Stevens and George, 2005). The components of DMA, such as collagen and fibronectin, also played important roles in promoting cell proliferation and reduced inflammatory response (Kang et al., 2012). Sebocytes also secreted more lipids on DMA-PCL scaffolds, contributing to continuous lipid secretion to the ocular surface and help form the lipid layer of the tear film after transplantation. In the *in vivo* study, SZ95 sebocytes seeded on DMA-PCL scaffolds still proliferated well after 1 month under the skin of nude mice. This suggested that the scaffolds seeded with SZ95 sebocytes might be a promising biomaterial for the regeneration of tarsal plate defects.

SZ95 sebocytes were selected as promising seed cells in this study to restore the function of secreting lipids. The meibomian gland is a type of sebaceous gland located on the upper and lower tarsal plates with a tubular structure and a full secretory function. Meibocytes or sebocytes secrete lipids to the ocular or skin surface, and the lipid layer functions as a barrier (Chhadva et al., 2017). The tarsal plate has poor regenerative capacity due to the lack of vascular tissues around it. Therefore, the source of meibocytes is limited. With the absence of meibomian glands, many patients with eyelid defects develop dry eye disease. In contrast, sebocytes are widely distributed throughout the body, secreting lipids, triglycerides and free fatty acids to protect the skin. As sebocytes have lipid secretion function and they are rich in autologous sources, they may serve as important seed cells for the replacement of meibocytes in the future.

This study also had some limitations. First, the scaffolds were designed and printed according to the scale of the human tarsal plate. The thickness of the scaffold was relatively thick, making it difficult to repair the defect *in situ* in animals, such as rabbits or rats. Second, the sebocytes were seeded on the scaffolds after printing was completed; however, the accurate distribution of cells could not be guaranteed. The 3D bioprinting technology can be applied to print sebocytes and materials simultaneously in the future, for the accurate distribution of sebocytes in the acinar position and formation of a functioning acinus.

## Conclusions

This study was novel in using the 3D printing technology to print PCL scaffolds that simulated the human tarsal plate. Subsequently, DMA was coated over the scaffolds through cell culturing and decellularization of hADSCs. DMA coated on the scaffolds made the surface more hydrophilic. The DMA-PCL scaffolds significantly enhanced sebocyte adhesion and proliferation. Moreover, sebocytes could differentiate well on the scaffold and exert the function of secreting lipids. Compared with scaffolds without DMA, the DMA-PCL scaffolds significantly suppressed inflammatory gene and apoptosis gene expression. At the same time, sebocytes climbed out of the scaffolds and formed a meibomian gland acinar-like structure and an epithelial layer after 1 month *in vivo*. Based on these results, it was believed that the 3D-printed scaffolds, combined with the decellularization technology, could be largely applied to tissue engineering. The 3D-printed DMA-PCL scaffolds along with sebocytes are promising substitutes in tarsal plate tissue engineering.

## Data Availability Statement

The datasets generated for this study are available on request to the corresponding author.

## Ethics Statement

The animal study was reviewed and approved by Animal Ethics Committee of Shanghai Ninth People's Hospital affiliated to Shanghai Jiao Tong University School of Medicine.

## Author Contributions

LC, HS, and YF designed the study and the experiments. LC and DY performed the experiments. NW and WZ contributed to the data analysis. QY participated in the design of the experiments and the revision of the final article. CY, HS, and YF revised the manuscript. CZ established the human sebaceous gland cell line SZ95. All authors discussed the results, reviewed the manuscript, and approved the final version of the manuscript.

### Conflict of Interest

The authors declare that the research was conducted in the absence of any commercial or financial relationships that could be construed as a potential conflict of interest.

## Supplementary Material

Supplementary Video 1 Contact angle of DMA-PCL scaffolds.
