# Corrigendum: 3D-Printed Poly-Caprolactone Scaffolds Modified With Biomimetic Extracellular Matrices for Tarsal Plate Tissue Engineering

**DOI:** 10.3389/fbioe.2020.00548

**Published:** 2020-06-03

**Authors:** Liangbo Chen, Dan Yan, Nianxuan Wu, Weijie Zhang, Chenxi Yan, Qinke Yao, Christos C. Zouboulis, Hao Sun, Yao Fu

**Affiliations:** ^1^Department of Ophthalmology, Ninth People's Hospital, Shanghai Jiao Tong University School of Medicine, Shanghai, China; ^2^Shanghai Key Laboratory of Orbital Diseases and Ocular Oncology, Shanghai, China; ^3^Departments of Dermatology, Venereology, Allergology and Immunology, Dessau Medical Center, Brandenburg Medical School Theodor Fontane, Dessau, Germany

**Keywords:** 3D printing, decellularized matrix, PCL scaffold, sebocytes, tarsal plate

Christos C. Zouboulis was not included as an author in the published article. The corrected Author Contributions statement appears below. In addition, “BeNa Culture Collection, China” was cited instead of “Zouboulis et al. 1999.” A correction has been made to the Materials and Methods section, subsection Culture of SZ95 Sebocytes and Cell Morphology on 3D Scaffolds, paragraph 1:

“A total of 1 × 10^5^ SZ95 sebocytes (Zouboulis et al., 1999) were seeded on PCL scaffolds or DMA-PCL scaffolds, in DMEM (Gibco, CA, USA), supplemented with 10% FBS (Gibco), 5 ng/mL recombinant human epidermal growth factor (Peprotech, USA), and 100 U/mL penicillin/streptomycin (Gibco, USA) in a humidified atmosphere containing 5% CO_2_ at 37°C. The detailed cell culture method was the same as mentioned earlier for hADSCs. The medium was replaced every other day. Seven days after cell seeding, the scaffolds were fixed with 0.25% glutaraldehyde (Merck, Germany) at 4°C overnight. The samples were rinsed with PBS three times and then dehydrated with graded concentrations of ethanol (30, 50, 70, 80, 90, and 100% *v/v*) for 10 min each. Subsequently, the samples were critical-point dried, following which they were sputter-coated with gold and examined using an SEM.”

## Author Contributions

LC, HS, and YF designed the study and the experiments. LC and DY performed the experiments. NW and WZ contributed to the data analysis. QY participated in the design of the experiments and the revision of the final article. CY, HS, and YF revised the manuscript. CZ established the human sebaceous gland cell line SZ95. All authors discussed the results, reviewed the manuscript, and approved the final version of the manuscript.

The authors apologize for this error and state that this does not change the scientific conclusions of the article in any way. The original article has been updated.

## References

[B1] Zouboulis C. C. Seltmann H. Neitzel H. Orfanos C. E. (1999). Establishment and characterization of an immortalized human sebaceous gland cell line (SZ95). J Invest Dermatol. 113, 1011–1020. 10.1046/j.1523-1747.1999.00771.x 10594745

